# Deep Vein Thrombosis in a Patient With Thalassemia Minor: A Case Report

**DOI:** 10.1002/ccr3.71497

**Published:** 2025-11-23

**Authors:** Alka Basnet, Bimarsh Acharya, Mausami Shrestha, Saroj Thapa

**Affiliations:** ^1^ KIST Medical College and Teaching Hospital Lalitpur Uttar Pradesh India; ^2^ Watford General Hospital Watford UK

**Keywords:** case report, deep vein thrombosis, hemoglobin, thalassemia minor

## Abstract

Deep vein thrombosis may develop in patients with beta‐thalassemia minor despite its benign hematologic course. Clinicians should consider thrombotic risk in these patients, ensuring timely recognition and management to prevent potentially life‐threatening complications.

## 1 Introduction

Thalassemia comprises inherited disorders of hemoglobin synthesis resulting in variable degrees of anemia. Beta‐thalassemia minor is generally asymptomatic and often discovered incidentally, whereas major forms require lifelong transfusions [1]. Although thromboembolic complications are well recognized in beta‐thalassemia intermedia and major, their occurrence in beta‐thalassemia minor is rarely reported [2]. Recent studies suggest that hypercoagulability in thalassemia may arise from chronic platelet activation, endothelial dysfunction, and red cell membrane abnormalities [2, 3]. However, the precise mechanisms and clinical significance in minor variants remain unclear.

We report a rare case of deep vein thrombosis in a patient with beta‐thalassemia minor, highlighting the need for increased awareness of thrombotic risk even in clinically mild forms of the disease.

## 2 Case History/Examination

A 40‐year‐old woman with no significant past medical history presented to the emergency department with swelling and pain of the left lower limb for 3 days. She denied recent trauma, surgery, or prolonged immobilization. There was no history of bleeding, blood transfusion, or hormonal therapy.

On examination, she appeared pale but hemodynamically stable. Local examination revealed diffuse swelling and tenderness of the left leg with a weak dorsalis pedis pulse. There was no erythema or ulceration.

## 3 Differential Diagnosis, Investigations and Treatment

Laboratory findings showed hemoglobin (Hb) 60 g/L (reference range: 120–155 g/L), mean corpuscular volume (MCV) 66 fL (80–96 fL), and packed cell volume (PCV) 0.19 L/L (0.36–0.46 L/L), indicating microcytic hypochromic anemia. Total leukocyte and platelet counts were within normal limits. Peripheral smear demonstrated microcytic, hypochromic red cells. Reticulocyte count was 0.4% (0.5%–2.5%), serum iron 4.8 μmol/L (11–30 μmol/L), total iron‐binding capacity (TIBC) 82 μmol/L (45–81 μmol/L), and transferrin saturation 5.8% (15%–50%).

**FIGURE 1:**
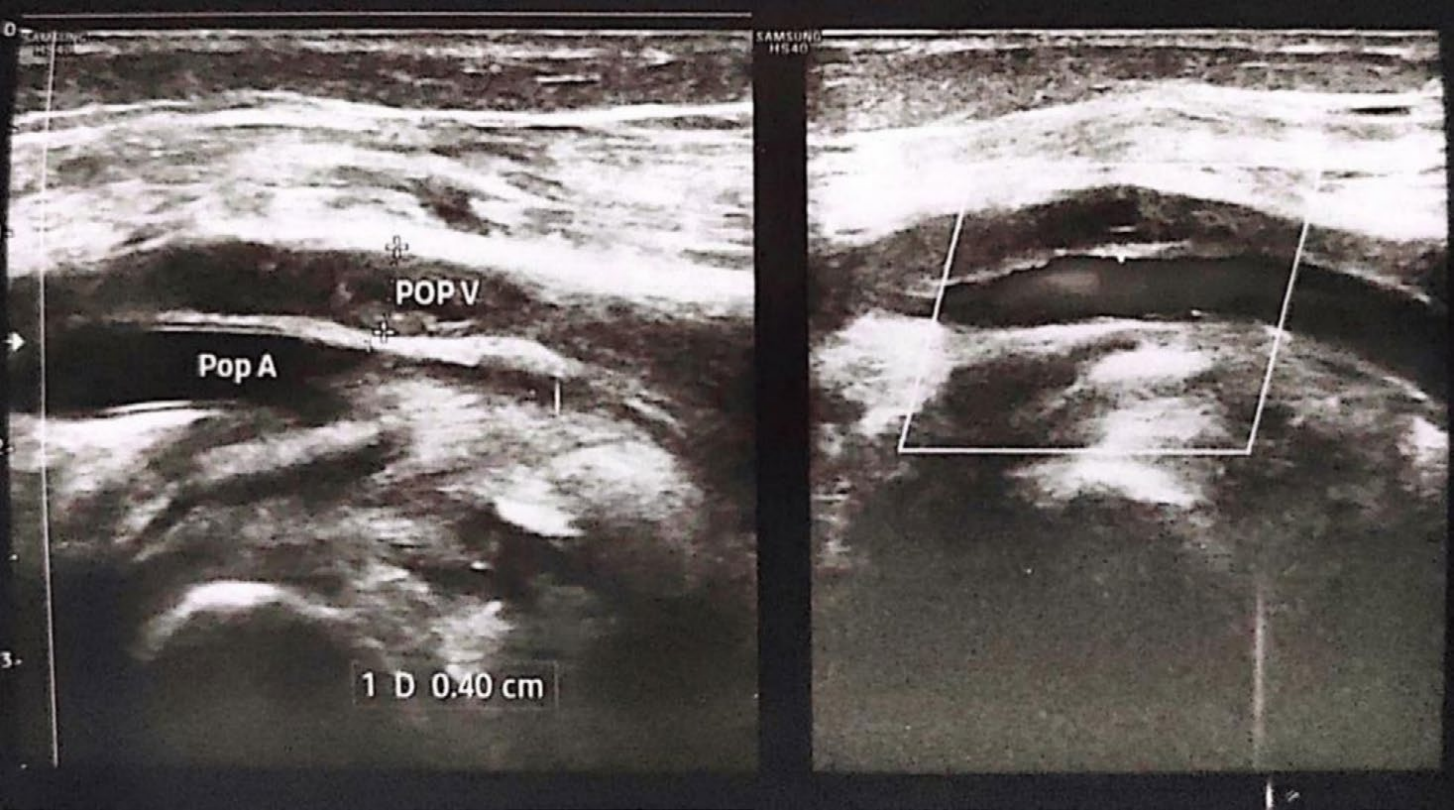
Duplex ultrasonography of the left lower limb showing echogenic thrombus causing luminal dilation and non‐compressibility of the popliteal vein, with complete occlusion of the common femoral vein and thrombus extension into the superficial femoral vein.

Hemoglobin electrophoresis revealed elevated HbA levels consistent with beta‐thalassemia minor (Table 1). Coagulation studies showed activated partial thromboplastin time 80 s (26–36 s) and D‐dimer > 15 mg/L (reference < 0.5 mg/L). Duplex ultrasonography demonstrated non‐compressibility of the left femoral and popliteal veins with absent flow signal, confirming acute deep vein thrombosis (Figure 1).

**TABLE 1:** Showing the results of Hb electrophoresis suggestive of beta thalassemia trait.

Parameter	Patient value	Range
HbF	1.3%	< 1%
HbA0	77.3%	95%–98%
HbA2	4.5%	< 3.5%
D‐dimer	> 15 μg/mL	< 0.5 μg/m

The patient was admitted to the high‐dependency unit. She received three units of packed red blood cells and oral iron supplementation. Following stabilization of hemoglobin, anticoagulation with intravenous heparin was initiated and later transitioned to oral apixaban on discharge. The patient remained stable and was advised regular hematologic follow‐up.

## 4 Discussion

Beta‐thalassemia minor is the least‐studied thromboembolic risk factor. However, hypercoagulability associated with thromboembolic events has been found to contribute significantly to morbidity and mortality among patients with beta‐thalassemia. Several studies have revealed increased platelet activation levels in beta‐thalassemia intermedia and splenectomized thalassemia patients compared with normal individuals [2]. Chenpeng et al. documented elevated levels of prothrombin fragment 1 + 2 (F1 + 2), an indicator of a hypercoagulable state, in beta‐thalassemia/HbE patients compared to healthy individuals. Increased platelet activation (CD41 + CD62P+) was found abundantly in both non‐splenectomized and splenectomized beta‐thalassemia/HbE patients. Furthermore, they suggested that higher platelet activation, hypercoagulation, and high serum ferritin levels, along with the upregulation of platelet proteins, are present in non‐splenectomized patients. Thus, coagulation monitoring is warranted in this group of patients.

The expression of CD62P, which mediates platelet–leukocyte adhesion through P‐selectin glycoprotein ligand 1 (PSGL‐1), was also found to be increased. These interactions promote thrombin generation, thereby contributing to the hypercoagulable state observed in beta‐thalassemia.

Venous thromboembolism (VTE), including deep vein thrombosis (DVT) and its potentially fatal complication, pulmonary embolism, is a major contributor to the global disease burden [3]. Thrombosis and embolism arise from the interaction between blood stasis, venous wall injury, and hypercoagulability (Virchow's triad) and can also be influenced by genetic predisposition [4]. Multiple factors contributing to vessel occlusion and chronic activation of the coagulation system are present in patients with beta‐thalassemia. The hypercoagulable state in thalassemia has been linked to abnormal red blood cells, increased platelet aggregation, chronically activated platelets, splenectomy, and augmented prothrombin generation. Winichagoon et al. [5] illustrated that chronic platelet activation and enhanced platelet aggregation are evident in thalassemia patients, confirmed by increased expression of CD62P (P‐selectin) and CD63.

A high incidence of thromboembolic events has been reported primarily in beta‐thalassemia intermedia and thalassemia major. Venous events are more frequent in thalassemia intermedia, while arterial events predominate in thalassemia major [6]. In a multicenter study, 32 of 735 patients (4.35%) presented with thromboembolic events, with incidences of 3.95% among 685 beta‐thalassemia major patients and 9.61% among 52 beta‐thalassemia intermedia patients [7]. Another study reported an overall thrombotic complication rate of 5.3% among 495 thalassemia patients with a median age of 28 years. Among them, thromboembolic events occurred in 3.3% of the 421 beta‐thalassemia major patients and in 16.2% of the 74 patients with beta‐thalassemia intermedia [8]. Similarly, a 10‐year follow‐up of 83 patients with beta‐thalassemia intermedia found that 24 (29%) developed DVT, pulmonary embolism, or portal vein thrombosis, with recurrent VTEs occurring in nine cases [9].

Previous studies have demonstrated significant platelet–monocyte and platelet–neutrophil aggregation, as well as altered platelet proteomes, in beta‐thalassemia/HbE patients, consistent with a hypercoagulable state [10]. These findings indicate that platelet–leukocyte interactions may enhance thrombin formation, contributing to thrombogenesis in thalassemia. Moreover, platelet aggregation proteins such as Hsp70, protein disulfide‐isomerase, eukaryotic translation initiation factor 5A‐1, peroxiredoxin‐2, and superoxide dismutase Cu‐Zn were abundantly expressed in beta‐thalassemia/HbE.

Red blood cells from thalassemia major (TM) and intermedia (TI) patients have been shown to adhere to cultured endothelial cells and express adhesion molecules and tissue factors in circulation, accompanied by decreased levels of protein C and protein S compared with healthy individuals [11]. Consistently, Eldor et al. [12] found a shortened platelet lifespan in beta‐thalassemia major and intermedia compared with healthy individuals (107 ± 36 h vs. 248 ± 51 h) after splenectomy. The mean platelet lifespan in non‐splenectomized thalassemia patients was 102 ± 64 h compared with 224 ± 23 h in healthy individuals (*p* < 0.01). These findings suggest increased platelet consumption or morphologic abnormalities, similar to those observed in chronic thrombotic conditions such as diabetes mellitus, severe atherosclerosis, and other hypercoagulable states.

While beta‐thalassemia intermedia and major, as well as post‐splenectomy patients, are known to be highly vulnerable to thrombosis, this case adds to the literature by suggesting that beta‐thalassemia minor may also predispose individuals to serious thrombotic or embolic complications, underscoring the need for clinical awareness and early intervention.

## 5 Conclusions

Thrombosis is mainly an episodic complication related to a temporary activation of hemostasis. Thalassemia has been well‐documented in association with hypercoagulability or thrombosis. Venous thrombosis is more prevalent in beta thalassemia, splenectomy, and transfusion‐naive patients. There are few cases reporting beta thalassemia minor, which is associated with venous thrombosis. We suggest possible approaches to establish an optimal strategy for preventing the occurrence of this complication in thalassemia minor.

## Author Contributions

**Alka Basnet:** conceptualization, investigation, supervision, writing – original draft, writing – review and editing. **Bimarsh Acharya:** methodology, validation, writing – original draft. **Mausami Shrestha:** formal analysis, funding acquisition, project administration, validation, writing – review and editing. **Saroj Thapa:** project administration, supervision, validation, visualization, writing – review and editing.

## Funding

The authors have nothing to report.

## Ethics Statement

The authors have nothing to report.

## Consent

Written informed consent was obtained from the patient for the publication of this case report and any accompanying images. A copy of the written consent is available for review by the editor‐in‐chief of this journal on request.

## Conflicts of Interest

The authors declare no conflicts of interest.

## Data Availability

Data sharing not applicable to this article as no datasets were generated or analyzed during the current study.
